# CDC20 Regulates the Progression of Clear Cell Renal Cell Carcinoma via the Wnt/β‐Catenin Signaling Pathway

**DOI:** 10.1155/genr/1270981

**Published:** 2026-07-19

**Authors:** YuHu Hao, Leizuo Zhao, Yanning Sun, Fan Peng, Tingting Xu, Wentao Deng, Qinghua Xia

**Affiliations:** ^1^ Department of Urology, Shandong Provincial Hospital, Shandong University, Jinan 250021, China, sdu.edu.cn; ^2^ Department of Urology, Dongying People’s Hospital, Dongying 257000, China, dysrmyy.com/; ^3^ Department of Urology, Shandong Provincial Hospital Affiliated to Shandong First Medical University, Jinan 250021, China, sph.com.cn

**Keywords:** ccRCC, CDC20, metastasis, proliferation, Wnt signaling pathway

## Abstract

**Objective:**

To detect the expression level of cell division cycle 20 homolog (CDC20) in clear cell renal cell carcinoma (ccRCC) and to study the biological function of CDC20 in ccRCC.

**Methods:**

CDC20 expression levels and clinical significance of ccRCC were determined using the Cancer Genome Atlas (TCGA) database. We detected the expression level of CDC20 in ccRCC with the help of immunohistochemistry (IHC). The effect of CDC20 on the proliferation of renal cancer cells was investigated using EDU and CCK‐8 proliferation assays. We used wound healing assays and Transwell assays to determine the effects of CDC20 on renal cancer cell migration and invasion and Wnt signaling pathway agonists in recovery experiments. The mechanism of CDC20 in ccRCC was detected using western blotting. Finally, the effect of CDC20 on renal cancer cells was verified in vivo in a nude mouse xenograft model.

**Results:**

Bioinformatics analysis found that CDC20 was upregulated in ccRCC, and its high expression was associated with poor prognosis in kidney renal cell carcinoma (KIRC) patients. In addition, we detected high expression of CDC20 in KIRC tissues, consistent with the results of bioinformatics analysis. Besides, knockdown of CDC20 can inhibit the biological functions of renal cancer cells. The recovery experiment proved that the biological function of the originally inhibited renal cancer cells was restored. In vivo, low expression of CDC20 can inhibit tumor growth.

**Conclusions:**

As CDC20 is highly expressed in KIRC tissues, it can be used as both a therapeutic target and a prognostic marker.

## 1. Introduction

The International Agency for Research on Cancer (IARC) estimates that in 2022, there were 434,419 new cases of kidney cancer and 155,702 died of it, posing a serious threat to human life and health [1]. Renal cell carcinoma is the main type of kidney cancer. However, its pathogenic mechanism is still unclear, and the pathogenesis of renal cell carcinoma may be related to obesity and environmental and genetic factors [2]. There are three common pathological types of renal cell carcinoma, but the clear cell renal cell carcinoma (ccRCC) is the main pathological type of renal cell carcinoma [3]. Currently, most treatment methods for renal cell carcinoma are surgery, but after surgery, patients will still experience recurrence or distant metastasis [4, 5]. Therefore, searching for new molecular markers is particularly necessary for diagnosing and treating patients with advanced renal cancer.

The cell division cycle 20 homolog (CDC20) acts as a cell cycle checkpoint control factor whose primary function is to bind CDH1 and activate the anaphase‐promoting complex (APC) [6, 7]. Previous studies have found that abnormal expression of CDC20 also plays an essential role in the process of cell mitosis [8]. Recent studies have found that CDC20, a tumor‐promoting gene, plays an essential role in the occurrence and development of human tumors, while CDH1, a tumor suppressor, plays a key role in tumor proliferation and metastasis [9, 10]. Relevant literature shows that abnormal overexpression of CDC20 in different types of human tumors is associated with poor patient prognoses, such as hepatocellular carcinoma, prostate cancer, bladder cancer, breast cancer, and colorectal cancer [11, 12]. In kidney renal cell carcinoma (KIRC), CYP1B1 can inhibit the expression level of DAPK1, thereby upregulating the expression of CDC20 and promoting the biological functions of renal cell carcinoma [13]. Besides, inhibiting the expression of CDC20 can inhibit the activity of the Wnt/β‐catenin signaling pathway, induce cell cycle arrest, and further inhibit the proliferation and metastasis of tumor cells [14]. However, it is not yet clear whether CDC20 plays a similar role in ccRCC, and the mechanism by which it regulates ccRCC is worth further exploration.

Different from previous studies on ccRCC, our study revealed for the first time that downregulation of CDC20 can inhibit the expression levels of related proteins in the Wnt signaling pathway, such as β‐catenin, c‐myc, and cyclin D1. That is, inhibiting CDC20 expression can inhibit the Wnt signaling pathway, thereby inhibiting the biological functions of KIRC. This is of great significance for elucidating the role of CDC20 in ccRCC. As an oncogene, CDC20 plays an essential biological function, making it a promising therapeutic target and prognostic marker for renal cell carcinoma.

## 2. Materials and Methods

### 2.1. KIRC Tissue Samples

As long as patients have informed consent in writing, 20 human renal cancer tissue and normal renal tissue specimens were gathered from Dongying People’s Hospital between 2020 and 2022. For immunohistochemistry (IHC), paraffin‐embedded specimens were sliced. The ethics committee made efforts to obtain ethical approval for the study of Dongying People’s Hospital. The World Medical Association approved protocol was followed in the research.

### 2.2. Cell Lines, Antibodies, and Drugs

The cell lines used in our experiment were purchased from the Cell Bank of the Chinese Academy of Sciences, and the antibodies used in the experiments included rabbit anti‐CDC20 (Abcam, ab26483 1:1000), anti‐c‐myc (Abcam, ab32072 1:1000), anti‐E‐cadherin (Abcam, ab1416 1:1000), anti‐Vimentin (Abcam, ab92547 1:1000), anti‐N‐cadherin (Abcam, ab76011 1:1000), anti‐β‐catenin (Abcam, ab32572 1:1000), anti‐cyclin D1 (Abcam, ab134175 1:1000), anti‐β‐actin (Abcam, ab8226 1:1000), and anti‐c‐myc (Abcam, ab32072 1:1000) antibodies and Wnt agonist 1 (C22H25N3O2, M 363.45, MCE) (Shanghai, China).

### 2.3. Bioinformatics Analysis

We used three online tools, UALCAN [15], Human Protein Atlas (HPA) [16], and Gene Expression Profiling Interactive Analysis (GEPIA) [17] to analyze the expression of CDC20 in pan‐cancer and its association with clinicopathological characteristics of renal cancer patients’ sex. Next, we used the TCGA database, which can help us to know about the mRNA expression data for the KIRC part. The R language graphics analysis package was used to draw relevant statistical charts, and the data contained normal kidney samples and tumor samples. We used GENE set enrichment analysis (GSEA) to analyze whether CDC20 is connected with the Wnt/β‐catenin signaling pathway.

### 2.4. IHC

First, sections were cut from paraffin‐embedded tissue specimens and then processed through dewaxing and gradient alcohol and antigen retrieval processes. Then, the specimens on the sections were incubated with the CDC20 antibody overnight at 4°C. The samples were incubated with the secondary antibody for 30 min at room temperature on the next day, the expression of CDC20 was detected using a DAB kit (Abcam), and the nuclei were stained with hematoxylin (Abcam). Finally, an *H* score was calculated based on the assessment of two independent pathologists.

### 2.5. qRT‐PCR Assay

Total RNA from tissues or cells was extracted with Takara reagent (Takara). RNA was then subjected to concentration determination and reverse transcribed to cDNA. The qRT‐PCR conditions were preincubation at 95°C for 30 s, amplification at 95°C for 5 s and 60°C for 30 s, a total of 45 cycles. The primer sequences are CDC20 forward, 5′‐GGCACCAGTGATCGACACATTCGCAT‐3′ and reverse, 5′‐GCCATAGCCTCAGGGTCTCATCTGCT‐3′.

### 2.6. Western Blot Assay

Total protein was obtained from the cell line, and then the protein was denatured, electrophoresed, membrane transferred, sealed with skim milk, and washed with the primary antibody overnight. The membrane should be incubated for 1 h at room temperature with the secondary antibody before developing.

### 2.7. Establishment of CDC20 Low‐Expressing Cell Lines

To establish a renal cancer cell line with stable low expression of CDC20, we designed a CDC20 lentiviral vector from Genechem (Shanghai, China). We seeded two human renal carcinoma cell lines into 24‐well plates until the cell confluency reached about 50%, and a certain amount of lentivirus and polypropylene (10 μg) was added. The culture medium in the 24‐well plate was changed after 16 h, and then 2 μg/mL of puromycin (Sigma) was added. After 72 h, the transfection efficiency of the renal cancer cell lines was tested to establish two renal cancer cell lines with stable low expression of CDC20.

### 2.8. Cell Counting Kit‐8 Assay

In the experimental and control groups, cells were cultured and seeded in 96‐well plates in five replicate wells. Incubation of the cells with CCK‐8, followed by cultivation at 37°C for 1 h, was done with a reader after a certain period of time. We used a microplate to measure the absorbance at 450 nm.

### 2.9. EDU Cell Proliferation Assay

We used the EDU Cell Proliferation Assay Kit (RN: R11053.9) according to the manufacturer’s instructions. We designed the control and experimental groups, seeded 4000–5000 cells per well in a 96‐well plate, and cultivated to reach the normal growth stage. The instructions require that the cells be labeled with EDU, cell immobilized, Apollo stained, DNA stained, and finally observed under a fluorescence microscope.

### 2.10. Wound Healing Assay

Cells from human renal cell cancer were seeded in six‐well plates. A 200‐μl sterile pipette was used to scrape off the cell layer once 95% cell density had been reached. Then, serum‐free medium was added and the cells were cultivated for a specific time. Photographs were taken using a microscope, wound healing measurements were performed with ImageJ, and statistical graphs were drawn.

### 2.11. Transwell Migration and Invasion Assays

One day in advance, the Matrigel gel (Corning) stored at −20°C was thawed at 4°C. An appropriate proportion of gel and serum‐free medium was mixed, and the mixed gel was applied to the bottom of the chamber, which was placed at 37°C in an incubator for 3 h. Next, in this experiment, logarithmically growing renal cancer cells were digested, counted, and seeded evenly at the bottom of a chamber. The chamber was then placed in a medium containing 20% FBS. After 24 h, the Transwell chamber was taken out, the remaining cells and gel in the chamber were wiped with a cotton wipe, washed with PBS, stained, observed, and counted under a microscope, and finally, the corresponding statistical chart was drawn. Cell migration and invasion experiments differed only in that Matrigel was not used in migration experiments, and the regional steps were identical.

### 2.12. Xenografts

The ACHN cells in the logarithmic growth phase were made into a cell suspension with a concentration of 4 × 107/mL, and then the cell suspension with a concentration of 4 × 107/mL was vaccinated subcutaneously into the axilla of nude mice and then measured every 3 days. After a change in tumor volume, the mice were sacrificed after 35 days of feeding, and the tumor was excised for corresponding experiments and evaluations.

### 2.13. Statistics

We used the PRISM software (GraphPad 8.0) to perform statistical analysis. Statistical significance of differences between groups was measured with *t* test and one‐way ANOVA, and the relationship between IHC and pathological parameters was tested with Pearson’s chi‐square. A *p* < 0.05 was considered statistically significant.

## 3. Results

### 3.1. Relationship Between the Expression Level of CDC20 in Pan‐Cancer and Renal Cancer’s Pathological Characteristics

CDC20 expression levels in pan‐cancer and renal cancer and their clinicopathological characteristics were investigated. We used the online database analysis on the UALCAN website, which revealed that the expression level of CDC20 was considerably increased in ccRCC. In addition, the expression in BLCA, BRCA, KIRP, CESC, CHOL, ESCA, COAD, HNSC, LIHC, LUAD, LUSC, and other tumors was also upregulated (Figure 1(a) and Figure S1a). Tumor stage and grade, patient age and gender, and KIRC subtype were associated with lymph node metastasis (Figure 1b–d and Figure S1b–e). Through analysis of the GEO database, we found that CDC20 was significantly highly expressed in tumor tissues (Figure S2a, b). We also found that the expression level of CDC20 was related to the combination of stages (*p* < 0.0001) (Figure 1f). Through univariate and multivariate Cox regression analyses, we found that M stage (distant metastasis), histologic grade (G3/G4), and age (> 60 years) were independent risk factors for patient survival. CDC20 expression level was associated with poor prognosis in the univariate analysis (Figure S2c). As a result of these results, KIRC patients were categorized as high‐risk (CDC20 high expression) or low‐risk based on the median risk score (Figure 1g, h), and the generated survival curves were validated using ROC curves (Figure 1i). The validation results found that the high‐risk group had a poor prognosis.

FIGURE 1The expression of CDC20 is upregulated in pan‐cancer and is related to the occurrence and development of clear cell renal cell carcinoma. (a and S1a) CDC20 expression in various tumors: red and blue represent tumor tissues and normal tissues, respectively. (b) The differences in CDC20 mRNA expression levels in normal and renal cancer tissues were analyzed using a database. (c, d, and S1b–e) The differential expression of CDC20 in clinicopathological features of different tumors. (e, f) Patients with renal cancer and the expression of CDC20 are associated with a higher survival rate. (g–i) The optimal cutoff values divided KIRC patients into high‐risk and low‐risk groups, and plotted Kaplan–Meier survival curves, using ROC curves to predict 1‐year, 3‐year, and 5‐year survival rates. ^∗^ *p* < 0.05; ^∗∗^ *p* < 0.01; ^∗∗∗^ *p* < 0.001.

**Figure:**
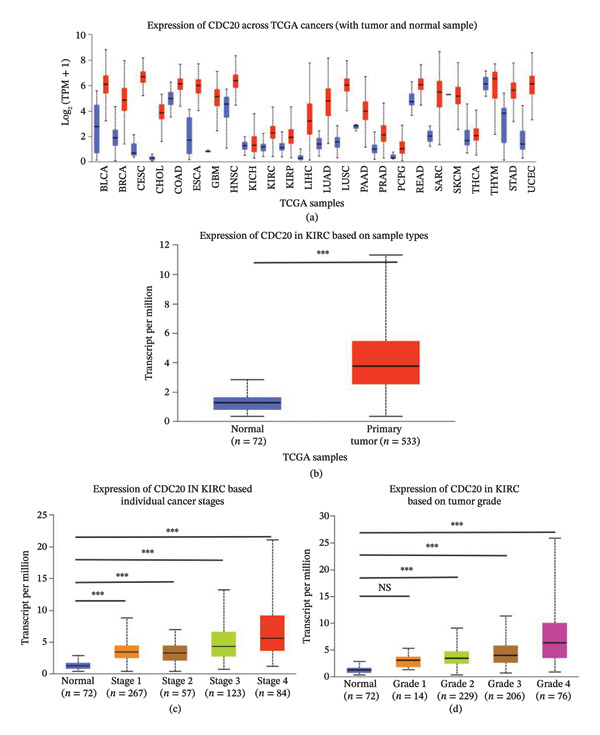

**Figure:**
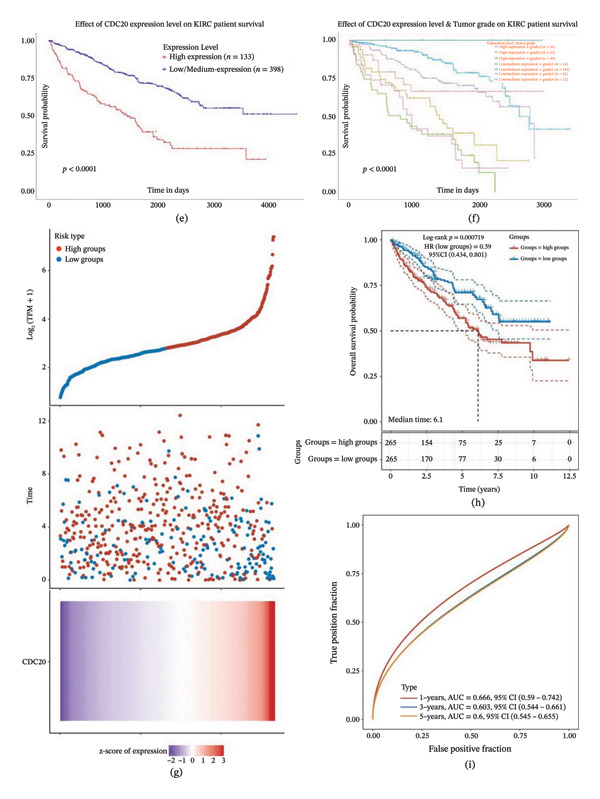

### 3.2. The Differential Expression of CDC20 in KIRC Tissues Is Confirmed by IHC and Quantitative Real‐Time PCR

**FIGURE 2:**
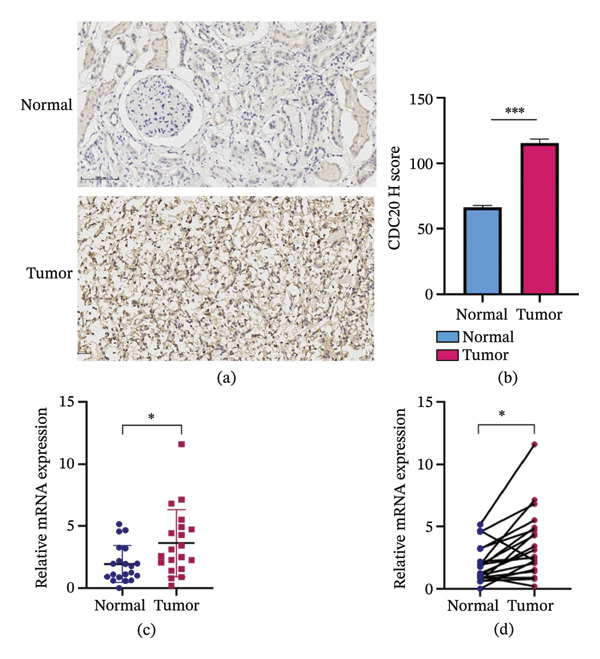
The expression of CDC20 in KIRC was assessed by qRT‐PCR and immunohistochemistry. (a and b) The gene expression of CDC20 in normal renal and renal cancer tissue was detected by immunohistochemistry, and the related statistics were drawn. (c and d) The mRNA expression levels of CDC20 in renal cancer tissues and normal renal tissues were detected by qRT‐PCR (Figure (d) shows paired tumor and matched adjacent nontumor tissues). ^∗^ *p* < 0.05; ^∗∗^ *p* < 0.01; ^∗∗∗^ *p* < 0.001.

CDC20 expression levels were determined using IHC and qRT‐PCR on 20 pairs of renal cancer tissues and adjacent tissues. Immunohistochemical detection revealed that the expression level of CDC20 was meaningfully higher in renal carcinoma tissues (Figure 2a, b). We then performed qRT‐PCR analysis on renal cancer tissues to detect CDC20 mRNA levels, and the results signified that CDC20 expression was higher in renal carcinoma tissues than in normal kidney tissues (Figure 2c, d). This is consistent with our immunohistochemical findings.

### 3.3. Establishment of Renal Cancer Cell Lines With Low Expression of CDC20 Through Screening and Culture

**FIGURE 3:**
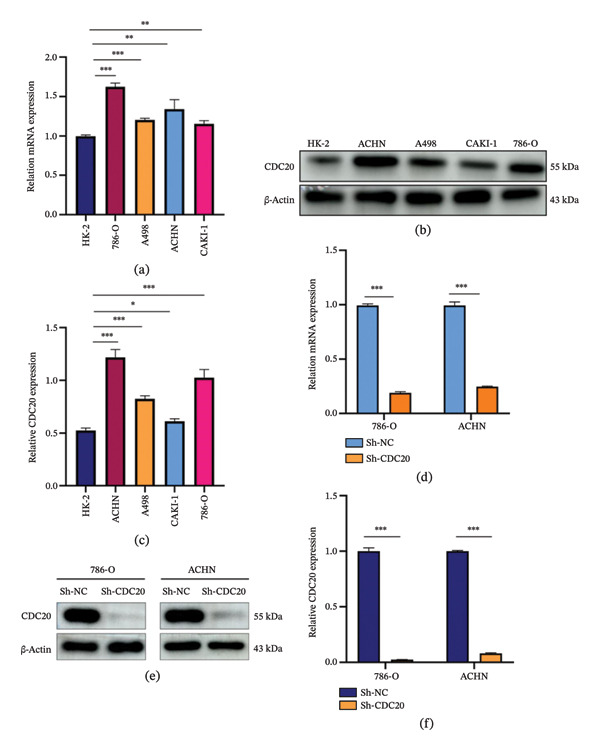
The expression level and expression effect of CDC20 in HK‐2, 786‐O, CAKI‐1, ACHN, and A498 cells. (a) qRT‐PCR detection of the expression level of CDC20 in HK‐2, 786‐O, CAKI‐1, ACHN, and A498 cells. (b, c) Western blot technique was used to detect the protein expression level of CDC20 in HK‐2, 786‐O, CAKI‐1, ACHN, and A498 cells and data analysis. (d) qRT‐PCR technique was used to detect the expression level of CDC20 in two cells. Knockdown efficiency in renal cancer cell lines. (e, f) Western blot technique detects the protein expression level and statistical analysis of CDC20 in two renal cancer cell lines.^∗^ *p* < 0.05; ^∗∗^ *p* < 0.01; ^∗∗∗^ *p* < 0.001.

To screen out suitable renal carcinoma cell lines for our study, qRT‐PCR assay and western blotting analysis were used to find CDC20 expression in HK‐2 cells and four renal cancer cell lines. The expression of CDC20 was higher than that of normal renal tubular epithelial cells, and more importantly, CDC20 expression was higher in 786‐O and ACHN than in CAKI‐1 and A498 (Figure 3a–c). Therefore, we chose 786‐O and ACHN as the cells for our subsequent experiments. To examine the biological functions of CDC20 in ccRCC, two cells expressing low levels of CDC20 were transfected using lentiviral technology. Renal carcinoma cell line was detected, and the transfection efficiency was revealed by quantitative real‐time PCR and western blotting (Figure 3d–f).

### 3.4. Inhibitory Effect of Low Expression of CDC20 on the Proliferation of 786‐O and ACHN Cells

**FIGURE 4:**
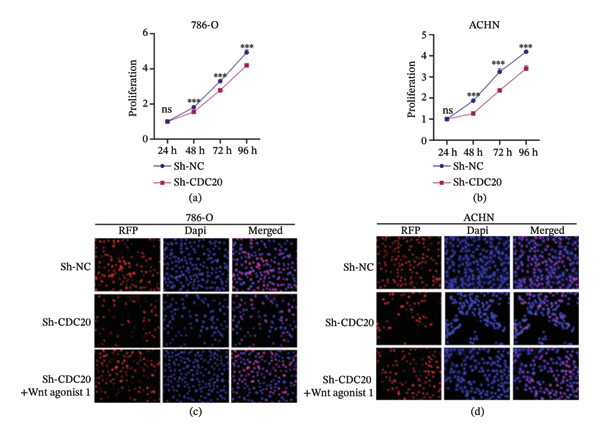
Cell proliferation experiments. (a and b) The OD values of 786‐O and ACHN cells in the Sh‐NC group and the Sh‐CDC20 group were determined by the CCK‐8 experiment, and the relative growth rate of the experimental group and the control group was calculated. (c, d) EDU cell proliferation assay was used to determine the proliferation numbers of 786‐O and ACHN cells in the Sh‐NC group, Sh‐CDC20, and Sh‐CDC20 + Wnt agonist 1 groups. ^∗^ *p* < 0.05; ^∗∗^ *p* < 0.01; ^∗∗∗^ *p* < 0.001.

An investigation was conducted to determine whether kidney cancer cells proliferate more rapidly when CDC20 expression is low; we used the CCK‐8 method (Figure 4a, b) and EDU cell proliferation tests (Figure 4c, d) to detect the cells in the control and experimental groups. It was found that renal cancer cell lines expressing low levels of CDC20 were less likely to proliferate.

### 3.5. A Knockdown of CDC20 Inhibited Tumor Cell Migration, Invasion, and Epithelial–Mesenchymal Transition (EMT) by Affecting the Wnt Signaling Pathway

Our experiments found that cell lines of renal cancer were susceptible to CDC20’s proliferating effect. As CDC20 was proved to contribute to the development of human cutaneous squamous cell carcinoma through the Wnt/β‐catenin signaling pathway [18], we found that CDC20 is indeed related to the Wnt signaling pathway through GSEA (Figure 5a). Therefore, in the following experiments, we mainly verified whether CDC20 could affect the biological function of renal cancer cells. In addition, we used agonists of the pathway to perform a rescue experiment. It was found by wound healing assay (Figure 5b–d) and Transwell experiments (Figure 5e–g) that the biological function of low‐expressing CDC20 renal cancer cell lines was significantly inhibited, and a Wnt agonist, however, restored the migration and invasion levels of the renal cancer cell lines that had previously been inhibited.

FIGURE 5Knockdown of CDC20 can inhibit the migration, invasion, and EMT of renal cell carcinoma, and the possible molecular mechanism was explored in this study. (a) GSEA database reveals the relationship between CDC20 and the Wnt signaling pathway. (b–d) Wound healing assay. (e–g) Transwell assay. (h–j) Western blot to detect CDC20, N‐cadherin, E‐cadherin, Vimentin, cyclin D1, β‐catenin, c‐myc, and β‐actin. (k–m) After knocking down CDC20, Wnt agonist 1 was added to detect the expression levels of cyclin D1, β‐catenin, c‐myc, and β‐actin. ^∗^ *p* < 0.05; ^∗∗^ *p* < 0.01; ^∗∗∗^ *p* < 0.001.

**Figure:**
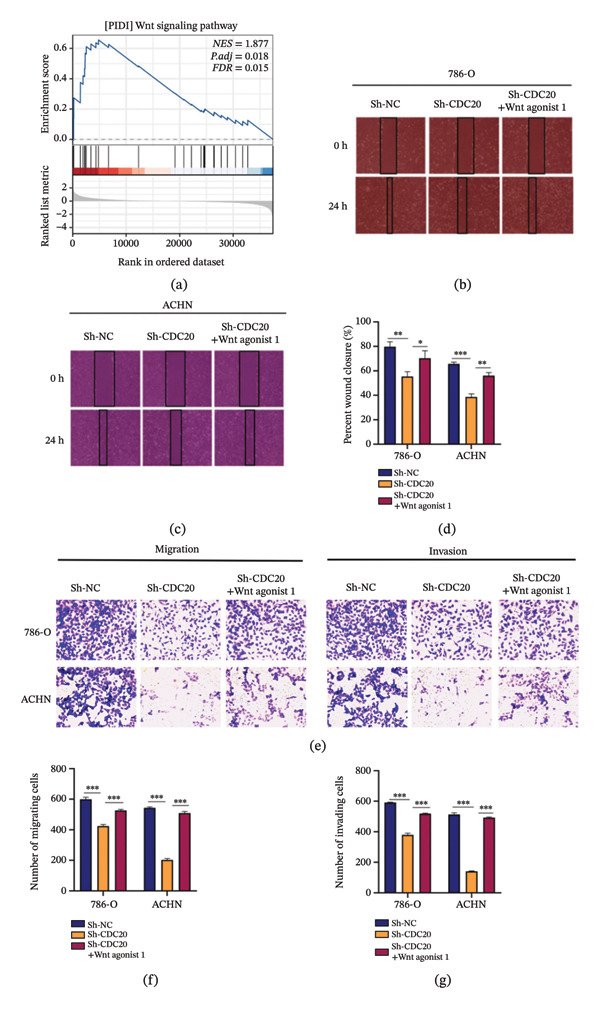

**Figure:**
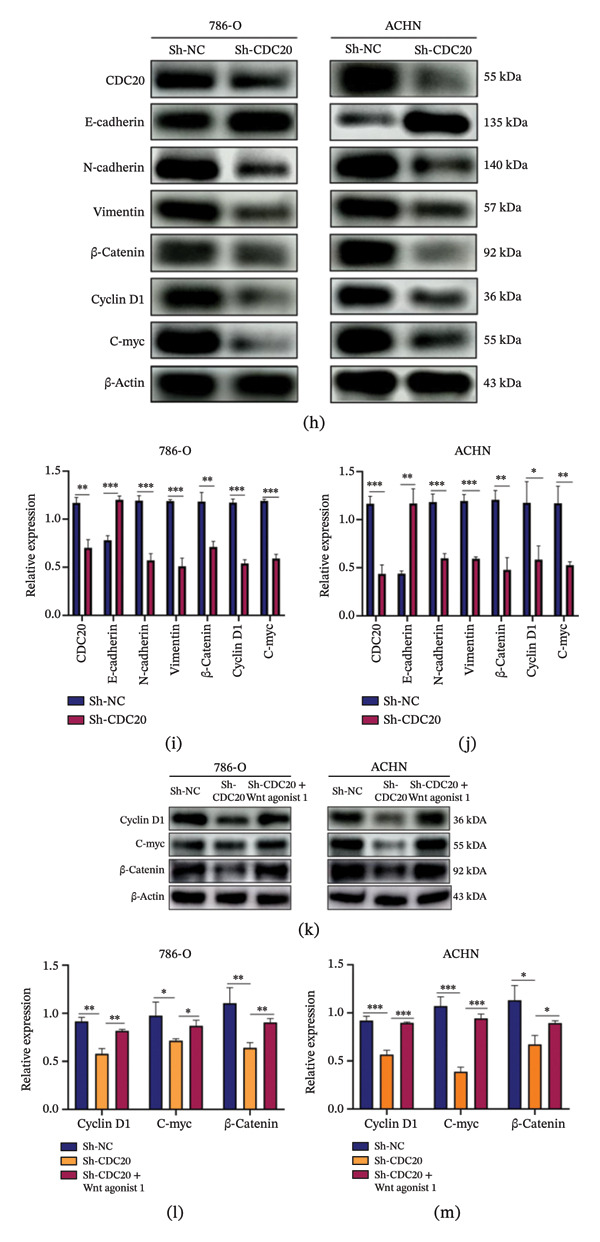

Through western blotting experiments, we found that low expression of CDC20 in two renal cancer cell lines resulted in a significant increase in the expression of E‐cadherin; however, the expression levels of N‐cadherin and Vimentin were considerably decreased. This indicates that renal carcinoma cell lines with low expression of CDC20 can inhibit EMT. Our experiments also found that renal carcinoma cell lines with low expression of CDC20 could suppress the expression levels of cyclin D1, β‐catenin, and c‐myc proteins (Figure 5h–j). With Wnt agonist 1, we conducted relevant rescue experiments to verify that CDC20 inhibits the Wnt signaling pathway. The addition of Wnt agonist 1 after knockdown of CDC20 restored the initially reduced the β‐catenin, cyclin D1, and c‐myc expression levels (Figure 5k–m). CDC20 expression is low in renal cancer cell lines, which inhibits the Wnt/β‐catenin pathway.

### 3.6. A Low Level of CDC20 Expression Inhibits the Growth of ACHN Cells in Mice

**FIGURE 6:**
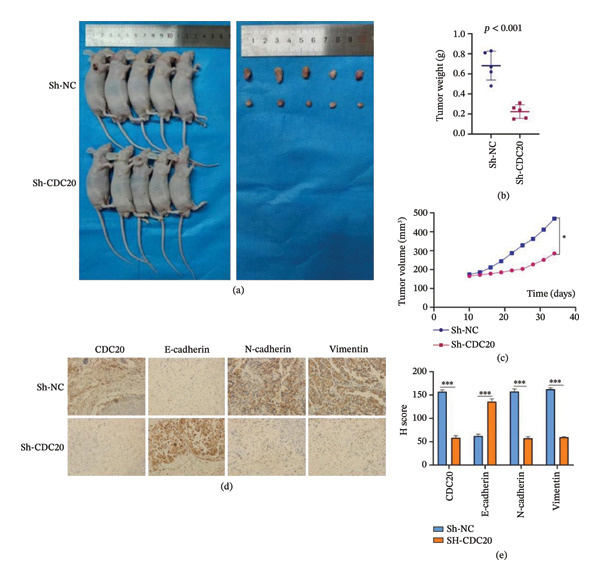
Knockdown of CDC20 can inhibit tumor growth in mice. (a) Tumor images in nude mice. (b, c) Tumor weight (*p* < 0.001) and tumor volume in the Sh‐NC group and the Sh‐CDC20 group. (d, e) The expression levels of CDC20, N‐cadherin, E‐cadherin, and Vimentin were detected by immunohistochemistry in nude mice. ^∗^ *p* < 0.05; ^∗∗^ *p* < 0.01; ^∗∗∗^ *p* < 0.001.

**FIGURE 7:**
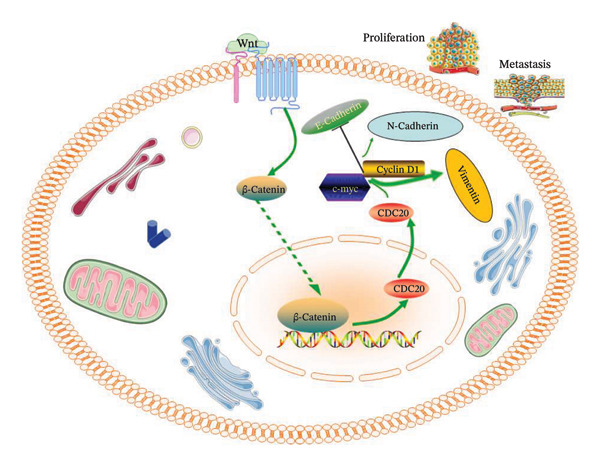
Schematic diagram of CDC20 affecting the biological function of clear cell renal cell carcinoma through the Wnt/β‐catenin signaling pathway (further experiments are required).

An experiment in nude mice was conducted to determine whether renal cancer cells with low CDC20 expression could inhibit tumor growth (Figure 6a). For our study, we used the ACHN cell line, which can help us do animal experiments. We revealed that the tumors in the low‐expressing CDC20 group were smaller in size and lower in weight (Figure 6b, c). Afterward, tumor tissue from mice was immunohistochemically analyzed. A CDC20 knockdown group’s tumor tissues expressed significantly lower levels of N‐cadherin, Vimentin, and CDC20 than the control group’s tissues. A considerable increase in E‐cadherin expression was detected in tumor tissues of the CDC20 knockdown group compared to the control group (Figure 6d, e). Through this experiment, we revealed that knockdown of CDC20 could restrain the proliferation, migration, and invasion of renal cancer cells. Besides, the underlying mechanism of CDC20 in ccRCC cells was also mapped (Figures 7).

## 4. Discussion

Globally, in the urogenital region, ccRCC is among the most common cancers with the highest mortality rate. Currently, there are many treatment methods for advanced metastatic RCC. Uncertainties and controversies in cell carcinoma‐related research still exist, and more importantly, the fatality rate of patients with advanced metastatic renal cancer is still high worldwide [19]. Molecular abnormalities are associated with renal cell carcinoma, according to previous research, including metabolomics, epigenetic modification, and transcriptomics [20, 21]. These studies show that ccRCC has significant molecular heterogeneity. Currently, the molecular mechanism of KIRC is still being studied, and these studies will help reveal the etiology and pathogenesis of renal cell carcinoma. Finding effective diagnostic and prognostic biomarkers may be critical in selecting patients who would benefit from active surveillance and other nonsurgical approaches.

CDC20 is a regulatory protein with pleiotropic biological functions. Its molecular structure contains seven highly conserved WD40 repeat domains, a structural feature that makes it a key regulatory molecule in the intracellular protein–protein interaction network [22]. Functionally, CDC20 is not only involved in the precise regulation of the cell cycle but also plays an important regulatory role in programmed cell death (apoptosis). As a core coactivator of the anaphase‐promoting complex/cyclosome (APC/C), CDC20 can specifically recognize and bind to characteristic sequence motifs in various substrate proteins, including degradation signals such as the activator box (A‐box), destruction box (D‐box), and Ken‐box. Through these molecular interactions, it precisely regulates the ubiquitination and degradation of target proteins, thereby achieving spatiotemporal control over the process of cell mitosis [23, 24]. Pathway studies indicate that CDC20 is involved not only in protein posttranslational modification, subcellular localization, and ubiquitination degradation processes, but also in the regulation of telomere length, gene transcription, and multiple key signaling pathways, including the Hippo pathway, the transforming growth factor beta (TGFβ) signaling axis, the beta‐catenin (β‐catenin)–mediated Wnt pathway, and the mitogen‐activated protein kinase (MAPK) cascade [25]. With the deepening of research on the molecular mechanisms of CDC20, a large body of clinical and basic research evidence indicates that dysregulation of CDC20 expression (particularly overexpression) is significantly associated with the occurrence, development, and metastasis of various human malignant tumors. More importantly, multiple large‐sample clinical studies have confirmed that high CDC20 expression levels are significantly correlated with shorter progression‐free survival (PFS) and overall survival (OS) in cancer patients, serving as an independent factor for poor prognosis. For example, in pancreatic ductal adenocarcinoma (PDAC), studies have found that CDC20 overexpression is positively correlated with clinical stage and lymph node metastasis, and the expression level of CDC20 can serve as an important reference indicator for predicting patient treatment response and survival prognosis [18]. In breast cancer, the expression level of CDC20 in breast cancer tissues is significantly higher than in normal breast tissues, and its overexpression is associated with significantly enhanced invasive characteristics of the tumor [26]. Research in non–small cell lung cancer (NSCLC) has shown that reducing CDC20 expression can effectively inhibit the abnormal proliferation of lung cancer cells by interfering with the normal function of the mitotic checkpoint, leading to tumor cell cycle arrest at the critical G2/M phase transition [27]. Furthermore, the expression level of CDC20 is positively correlated with the clinicopathological characteristics of patients with hepatocellular carcinoma. Relevant evidence suggests that in hepatocellular carcinoma cells with low CDC20 expression, the loss of endogenous PHD3 can significantly inhibit the decline of HIF‐1*α*, affect VEGF secretion, and consequently influence the biological functions of hepatocellular carcinoma [28]. However, the mechanism by which CDC20 regulates ccRCC has not yet been revealed.

Based on the current state of domestic and international research, to uncover the potential biological function of CDC20 in ccRCC, we analyzed the expression level and clinical significance of CDC20 in human renal carcinoma tissues using the TCGA database and the UALCAN website. The results showed that CDC20 expression was significantly higher in various cancer tissues, including renal carcinoma, compared to adjacent normal tissues, and high CDC20 expression was associated with a poorer prognosis for renal cancer patients. Based on this, we constructed a CDC20‐related prognostic risk model, which effectively revealed the differences in prognostic risk associated with different expression levels of the CDC20 gene. We observed that the use of bioinformatics is becoming increasingly important for detecting the role of related target proteins in ccRCC. Subsequently, we detected CDC20 expression at the molecular biology level using 20 pairs of collected renal cancer and adjacent tissue samples, as well as multiple renal carcinoma cell lines. The results showed that CDC20 expression was significantly upregulated at both the mRNA and protein levels in renal carcinoma tissues and cell lines, and this expression was correlated with clinicopathological features. To further elucidate the biological role of CDC20 in renal cell carcinoma, we constructed stable cell models with low CDC20 expression in two renal carcinoma cell lines, ACHN and 786‐O, which endogenously exhibit high CDC20 expression. Based on this, we explored the biological functions of the CDC20 gene in ccRCC cells. Using CCK‐8 and EdU cell proliferation assays, this study observed a significant decrease in the proliferative activity of both ACHN and 786‐O renal carcinoma cells following the inhibition of CDC20 gene expression. The study also confirmed through wound healing assays that CDC20 knockdown significantly impaired the wound closure process in ccRCC. Subsequent Transwell migration and invasion assays further verified that downregulating CDC20 expression significantly restricted the migration and invasion capabilities of ccRCC cells. Consistent with previous studies identifying the procancerous role of the CDC20 gene in other cancers, our experiments also confirmed that the CDC20 gene may be a key gene promoting the proliferation and progression of ccRCC and is associated with the malignant biological behavior of renal cancer.

During cancer development, EMT is generally considered a crucial step for tumor cells to acquire invasive and metastatic capabilities. EMT facilitates the detachment of tumor cells from the primary site and their colonization in distant organs. Activation of the canonical Wnt/β‐catenin signaling pathway can initiate the EMT program by increasing the expression of related transcription factors, thereby suppressing the expression of epithelial markers such as E‐cadherin while promoting the expression of mesenchymal markers like Vimentin and N‐cadherin. A substantial body of literature has clearly confirmed that the Wnt/β‐catenin signaling pathway, as a key intracellular regulatory network, plays a powerful role in the malignant biological behavior of tumor cells, including but not limited to cell proliferation, differentiation programs, and invasive and metastatic capabilities [29–31]. Further mechanistic studies have revealed that aberrant activation of this signaling pathway can trigger a cascade, leading to a significant upregulation in the transcription levels of downstream target genes, such as cyclin D1 and the proto‐oncogene c‐myc. The overexpression of these genes disrupts the normal balance of cell cycle regulation and enhances the invasive capacity of tumor cells, collectively promoting tumor proliferation, progression, and distant metastasis [32]. Accumulating evidence suggests that activation of the Wnt/β‐catenin signaling pathway is also involved in the development and progression of various other malignancies. Based on this molecular mechanism, reducing the expression of genes related to this pathway or using specific inhibitors targeting it has demonstrated significant antitumor effects in experimental studies, effectively blocking the abnormal proliferation, invasion, and metastasis of tumor cells [33]. For example, in cutaneous melanoma, β‐catenin forms a stable complex with transcription factors of the TCF/LEF family and translocates to the nucleus. This transcriptional activation complex can specifically bind to the promoter regions of cyclin D1 and c‐myc genes, significantly enhancing their transcriptional activity. The sustained overexpression of these downstream target genes disrupts the normal function of cell cycle checkpoints, accelerates the G1/S phase transition, and activates multiple prosurvival signaling pathways, collectively promoting the malignant transformation and tumor progression of melanoma cells [34]. Molecular analysis of esophageal squamous cell carcinoma has revealed that low expression of SALL4 inhibits the expression levels of genes associated with the Wnt/β‐catenin pathway, further suppressing the EMT capacity of tumor cells. This, in turn, inhibits cancer cell proliferation, migration, and invasion and reduces tumor growth rate and volume in a nude mouse xenograft model [35]. In colorectal cancer, cinobufagin inhibits the Wnt/β‐catenin signaling pathway in a concentration‐dependent manner, further suppressing the proliferation, migration, invasion, and EMT ability of tumor cells [36]. In ccRCC, highly expressed TRIM33 inhibits the occurrence and development of renal cancer cells by suppressing the Wnt/β‐catenin signaling pathway [37]. LGK974 inhibits PORCN expression, and the subsequent low expression of PORCN suppresses the activity of the Wnt‐activated pathway, thereby affecting the biological functions of renal cell carcinoma [38].

In this context, we first performed GSEA on ccRCC samples from the TCGA database to identify associated pathways. The results showed that high CDC20 expression was significantly positively correlated with the activation of the Wnt signaling pathway. Further western blot experiments revealed that in the two renal carcinoma cell lines with CDC20 knockdown, the expression level of the epithelial marker E‐cadherin was significantly increased, while the expression levels of the mesenchymal markers Vimentin and N‐cadherin were significantly decreased. This indicates that EMT is inhibited in renal carcinoma cell lines with low CDC20 expression, partially explaining why low CDC20 expression can inhibit the proliferation, migration, and invasion abilities of 786‐O and ACHN cells. However, upon the addition of a Wnt signaling pathway agonist, the biological functions of ccRCC cells and the expression levels of related molecules within the Wnt signaling pathway were restored. Finally, in vivo experiments further confirmed that reducing CDC20 expression in ACHN renal carcinoma cells significantly inhibited the growth rate and volume of tumors in mice. In conclusion, according to our study, inhibiting CDC20 expression in ccRCC may exert an antitumor effect, and this mechanism is highly likely achieved through the suppression of the Wnt/β‐catenin pathway.

## 5. Conclusion

In our study, we discovered that the expression level of CDC20 was considerably increased in renal cell carcinoma, and high expression of CDC20 was related with poor prognosis in ccRCC. Low expression of CDC20 can inhibit the biological functions of renal carcinoma cells. There may be an inhibition of the biological activity of the Wnt/β‐catenin pathway in renal carcinoma cells with low expression of CDC20. The limitation of this study is that we did not profoundly analyze the regulatory mechanism of CDC20 in KIRC. Our study suggests that CDC20 may be a potential target and prognostic marker for renal cancer therapy.

**Nomenclature**
CDC20: Cell division cycle 20 homolog
TCGA: The Cancer Genome Atlas
APC: Anaphase‐promoting complex
BLCA: Bladder urothelial carcinoma
BRCA: Breast cancer
CESC: Cervical squamous cell carcinoma
CHOL: Cholangiocarcinoma
COAD: Colon adenocarcinoma
ccRCC: Clear cell renal cell carcinoma
ESCA: Esophageal carcinoma
HNSC: Head and neck squamous cell carcinoma
KIRC: Kidney renal cell carcinoma
KIRP: Kidney renal papillary cell carcinoma
LIHC: Liver hepatocellular carcinoma
LUSC: Lung squamous cell carcinoma

## Author Contributions

Qinghua Xia and YuHu Hao designed the research methods, performed the experiments, and analyzed the data. Leizuo Zhao and YuHu Hao participated in experiments and data collection. Yanning Sun, and Fan Peng collected tumor tissues. Wentao Deng and Tingting Xu drafted and revised the manuscript.

## Funding

This work was supported by the National Natural Science Foundation of China (Grant no. 82272813), Traditional Chinese Medicine Science & Technology Project of Shandong Province (Grant no. M‐2022036), and Medical and Health Technology Development Project of Shandong Province, China (Grant no. 202104050603).

## Disclosure

All authors have read and approved the final manuscript.

## Ethics Statement

The protocol was approved by the Ethics Committee of Shandong Provincial Hospital Affiliated to Shandong First Medical University (approval no. NSFC 2022‐333) and certifies that the study was performed in accordance with its relevant guidelines and regulations. The ethics committee stipulated that the maximum diameter of the nude mouse tumor should not exceed 1.5 cm, and we ensured that the maximum diameter of the animals in our experiments is less than 1.5 cm.

The ethics committee made efforts to obtain human ethical approval for this study of Dongying People’s Hospital (approval no. DYYX 2021‐122). The World Medical Association‐approved protocol was followed in the research. All patients in this study provided informed written consent.

## Conflicts of Interest

The authors declare no conflicts of interest.

## Data Availability

The data used to support the findings of this study are available within the article and from the corresponding author upon request. The bioinformatics analysis methods used in this study can be found on the corresponding websites, UALCAN (https://ualcan.path.uab.edu), Human Protein Atlas (https://www.proteinatlas.org), Gene Expression Profiling Interactive Analysis (https://gepia.cancer-pku.cn), and Gene Set Enrichment Analysis (https://www.gsea-msigdb.org).
